# SARS-CoV-2 S1 and N-based serological assays reveal rapid seroconversion and induction of specific antibody response in COVID-19 patients

**DOI:** 10.1038/s41598-020-73491-5

**Published:** 2020-10-06

**Authors:** Abdullah Algaissi, Mohamed A. Alfaleh, Sharif Hala, Turki S. Abujamel, Sawsan S. Alamri, Sarah A. Almahboub, Khalid A. Alluhaybi, Haya I. Hobani, Reem M. Alsulaiman, Rahaf H. AlHarbi, M.-Z.aki ElAssouli, Rowa Y. Alhabbab, Ahdab A. AlSaieedi, Wesam H. Abdulaal, Afrah A. Al-Somali, Fadwa S. Alofi, Asim A. Khogeer, Almohanad A. Alkayyal, Ahmad Bakur Mahmoud, Naif A. M. Almontashiri, Arnab Pain, Anwar M. Hashem

**Affiliations:** 1 Vaccines and Immunotherapy Unit, King Fahd Medical Research Center, King Abdulaziz University, Jeddah, Saudi Arabia; 2 Department of Medical Laboratories Technology, College of Applied Medical Sciences, Jazan University, Jazan, Saudi Arabia; 3 Medical Research Center, Jazan University, Jazan, Saudi Arabia; 4 Faculty of Pharmacy, King Abdulaziz University, Jeddah, Saudi Arabia; 5 Pathogen Genomics Laboratory, Division of Biological and Environmental Sciences and Engineering (BESE), King Abdullah University of Science and Technology, Thuwa, Saudi Arabia; 6 King Abdullah International Medical Research Centre, King Saud bin Abdulaziz University for Health Sciences, Ministry of National Guard Health Affairs, Jeddah, Saudi Arabia; 7 Department of Medical Laboratory Technology, Faculty of Applied Medical Sciences, King Abdulaziz University, Jeddah, Saudi Arabia; 8 Department of Biochemistry, Faculty of Science, King Abdulaziz University, Jeddah, Saudi Arabia; 9 Department of Biology, Faculty of Science, King Abdulaziz University, Jeddah, Saudi Arabia; 10 Infectious Diseases Department, King Abdullah Medical Complex, Jeddah, Saudi Arabia; 11 Infectious Diseases Department, King Fahad Hospital, Almadinah Almunwarah, Saudi Arabia; 12 Plan and Research Department, General Directorate of Health Affairs Makkah Region, Ministry of Health, Makkah, Saudi Arabia; 13 Department of Medical Laboratory Technology, University of Tabuk, Tabuk, Saudi Arabia; 14 College of Applied Medical Sciences, Taibah University, Almadinah Almunwarah, Saudi Arabia; 15 Center for Genetics and Inherited Diseases, Taibah University, Almadinah Almunwarah, Saudi Arabia; 16 Research Center for Zoonosis Control, Hokkaido University, Kita-ku, Sapporo, Japan; 17 Nuffield Division of Clinical Laboratory Sciences (NDCLS), University of Oxford, Oxford, UK; 18 Department of Medical Microbiology and Parasitology, Faculty of Medicine, King Abdulaziz University, Jeddah, Saudi Arabia

**Keywords:** Infection, Viral infection

## Abstract

As the Coronavirus Disease 2019 (COVID-19), which is caused by the novel SARS-CoV-2, continues to spread rapidly around the world, there is a need for well validated serological assays that allow the detection of viral specific antibody responses in COVID-19 patients or recovered individuals. In this study, we established and used multiple indirect Enzyme Linked Immunosorbent Assay (ELISA)-based serological assays to study the antibody response in COVID-19 patients. In order to validate the assays we determined the cut off values, sensitivity and specificity of the assays using sera collected from pre-pandemic healthy controls, COVID-19 patients at different time points after disease-onset, and seropositive sera to other human coronaviruses (CoVs). The developed SARS-CoV-2 S1 subunit of the spike glycoprotein and nucleocapsid (N)-based ELISAs not only showed high specificity and sensitivity but also did not show any cross-reactivity with other CoVs. We also show that all RT-PCR confirmed COVID-19 patients tested in our study developed both virus specific IgM and IgG antibodies as early as week one after disease onset. Our data also suggest that the inclusion of both S1 and N in serological testing would capture as many potential SARS-CoV-2 positive cases as possible than using any of them alone. This is specifically important for tracing contacts and cases and conducting large-scale epidemiological studies to understand the true extent of virus spread in populations.

## Introduction

In December 2019, a cluster of atypical pneumonia was reported in Wuhan City, the capital of Hubei province in China. The etiological agent was quickly identified as a novel coronavirus, subsequently named as severe acute respiratory syndrome coronavirus 2 (SARS-CoV-2), and identified as a cause of the Coronavirus Disease 2019 (COVID-19)^1^. Within weeks of its discovery, SARS-CoV-2 has rapidly spread to most countries around the world, causing large scale morbidity and mortality. Eventually, it was recognized as a pandemic by the World Health Organization (WHO) in early March of 2020. The rapid and continued spread of the virus has triggered the implementation of unprecedented public health measures by affected countries, including travel bans, border closures, enforced curfew, the lockdown of cities, and shutdown of most businesses, public gatherings, and other activities. Nevertheless, the spread of the virus was further complicated by the absence of vaccines and specific therapeutics to date, although Remdesivir and favipiravir (avifavir) have been conditionally approved in a few countries for limited use^2,3^.

Coronaviruses (CoVs) are a large group of viruses that can infect a wide range of hosts, including humans, animals, and birds^4^. They are classified into four genera; alpha, beta, gamma, and delta, in which only viruses from alphacoronaviruses (alpha-CoVs) and betacoronaviruses (beta-CoV) were recognized to infect humans so far^4^. SARS-CoV-2 belongs to the beta-CoV genus, which also contains two other highly pathogenic human CoVs; SARS-CoV and MERS-CoV as well as a number of animal CoVs^5^. Genome sequence analysis shows that SARS-CoV-2 shares nearly 79.5% identity with SARS-CoV and ~ 96% with bat SARS-like CoVs^1^. CoVs are enveloped viruses with a positive-sense, single-stranded, ~ 30 kb RNA genome, which contains at least 6 open reading frames (ORFs)^5^. The first two-thirds of the genome encodes for polyproteins: pp1a and pp1ab that are processed by viral and host proteases into 16 non-structural proteins (nsp1-16)^5,6^. The other third of the genome encodes the four main structural proteins (envelope (E), membrane (M), spike (S), and nucleocapsid (N) proteins) as well as other accessory proteins^5,6^.

As SARS-CoV-2 continues to spread around the globe, it is crucial to understand the duration and nature of mounted immunity in response to infection, which is not yet fully understood and is currently under investigation. Furthermore, the actual extent of the current global COVID-19 pandemic is not well known; therefore, serological assays are critically needed to shed light on all these unanswered questions. Here, we report the development and validation of multiple indirect ELISA-based serological assays that can be adapted and used by laboratories to determine the immune status of individuals for surveillance and epidemiological studies, as we have previously described for MERS-CoV^7,8^. Using sera derived from either COVID-19 confirmed patients or known non-infected healthy controls, we validated our ELISAs and determined their cut-off values, sensitivity, and specificity. We also showed that our assays had no cross-reactivity using sera with known positivity to MERS-CoV and other common CoVs. Our study shows that SARS-CoV-2 IgM or IgG specific antibodies for either SARS-CoV-2 S1 or N antigens can be detected virtually in all real-time polymerase chain reaction (RT-PCR) confirmed COVID-19 patients included in our study as early as one week after disease-onset. Antibodies levels sharply increased by week two, with IgG persisting through week four compared to IgM, which peaked by week 2 or 3 before declining as previously shown^9^.

## Material and methods

### Samples

A 100 serum samples from healthy controls collected before the COVID-19 pandemic with one positive control from a confirmed COVID-19 patient were used to determine the cut-off values for the developed indirect ELISAs. Another set of samples including eight SARS-CoV-2 and MERS-CoV seronegative samples, two MERS-CoV seropositive samples, and three SARS-CoV-2 seropositive samples were used to determine the cross-reactivity of the assays. A third cohort of pre-pandemic samples (n = 125) and RT-PCR confirmed COVID-19 patients (n = 52) including samples collected during the 1st week (n = 10), 2nd week (n = 23), 3rd week (n = 14) or 4th week (n = 5) of symptoms-onset were used to evaluate the developed ELISAs. Onset of symptoms was based on clinical histories as reported by patients upon their hospital admission. Samples were obtained from multi-ethnicity patients or donors aged between 24 and 75 years, residing in Saudi Arabia. All samples from COVID-19 patients were collected from individuals admitted to hospital based on meeting COVID-19 case definition as per the Saudi Ministry of Health (MOH) guidelines and confirmed by RT-PCR assay targeting the envelop (E) and RNA dependent RNA polymerase (RdRp) genes. All samples were anonymized and used based on ethical approvals obtained from the Unit of Biomedical Ethics in King Abdulaziz University Hospital (Reference No 245-20), the Institutional Review Board at the Ministry of Health, Saudi Arabia (IRB Numbers: H-02-K-076-0320-279 and H-02-K-076-0420-285), and the Global Center for Mass Gatherings Medicine (GCMGM) (No. 20/03A), with informed consent obtained from all participants. All methods and experiments were performed in accordance with the relevant guidelines and regulations.

### Recombinant proteins

Recombinant SARS-CoV-2 S1 subunit of the S protein (amino acids 1–685, expressed in mammalian HEK293 cells), MERS-CoV S1 subunit (amino acids 1–725, expressed in mammalian HEK293 cells), and full-length S proteins (expressed in baculovirus-insect cells) from hCoV-OC43, hCoV-NL63, hCoV-229E, and hCoV-HKU1 viruses tagged with histidine tag (His-tag) were purchased commercially (Sino Biological, China). Recombinant SARS-CoV-2 and MERS-CoV N proteins were expressed and purified from *Escherichia coli* BL21 (DE3) cells using a nickel-nitrilotriacetic acid (Ni-NTA) column according to the manufacturer's protocol and as previously described^7^. Positive fractions of N proteins were pooled, aliquoted, and stored at − 80 °C until used. SARS-CoV-2 proteins were confirmed by Western blot using anti-His tag antibodies as well as SARS-CoV-2 seropositive and seronegative human serum samples as previously described^7^.

### Indirect ELISA

Recombinant SARS-CoV-2 S1, MERS-CoV S1, or full-length S proteins from other human CoVs at a concentration of 1 μg/ml in phosphate-buffered saline (PBS) were used to coat 96-well high binding ELISA plates (Greiner Bio One, Monroe, NC) with 50 μl per well. Similarly, in-house produced SARS-CoV-2 and MERS-CoV N proteins were used to coat plates at a concentration of 4 μg/ml. All plates were coated for overnight at 4 °C, washed thrice with PBS containing 0.05% tween-20 (PBS‐T), and blocked with 5% skim milk in PBS-T buffer at 37 °C for 1 h. After blocking, plates were washed thrice and incubated with serum samples diluted at 1:100 in PBS‐T with 5% milk for 1 h at 37 °C. Plates were then washed three times again with PBS-T, incubated with HRP‐conjugated goat anti‐human IgG (H + L) or IgM antibodies (Jackson ImmunoResearch, West Grove, PA) for 1 h, washed again, and incubated with TMB (3,3′,5,5′-tetramethylbenzidine) substrate (KPL, Gaithersburg, MD) at 37 °C for 30 min. The reaction was terminated by adding 100 μl per well of the ELISA stop solution (0.16 M sulfuric acid). The absorbance was measured at 450 nm using the ELx808™ Absorbance Microplate Reader (BioTek, Winooski, VT).

### Sequence homology analysis

Alignment and sequence identity of SARS-CoV-2 S1 and N proteins with respected regions from other known human CoVs including SARS-CoV, MERS-CoV, hCoV-OC43, hCoV-NL63, hCoV-229E, and hCoV-HKU1 were performed using Geneious Prime version 2020.0.3 (Geneious, Inc.) and heatmaps were created with Morpheus (https://software.broadinstitute.org/morpheus). The IDs of the used sequences are as follows: SARS-CoV-2 S1 (NCBI accession # YP_009724390.1) and N (NCBI accession # YP_009724397.2), SARS-CoV S1 (UniProt # P59594) and N (UniProt # P59595), MERS-CoV S1 (UniProt # W6A028) and N (UniProt # R9UM87), hCoV-OC43 S1 (UniProt # P36334) and N (UniProt # P33469), hCoV-NL63 S1 (UniProt # Q6Q1S2) and N (UniProt # Q6Q1R8), hCoV-229E S1 (UniProt # P15423) and N (UniProt # P15130-1), and hCoV-HKU1 S1 (UniProt # Q0ZME7) and N (UniProt # Q5MQC6).

### Statistical analysis

The sensitivity of each ELISA was determined as (the number of samples that are true positives/the total number of samples that are true positives and false negatives × 100), and the specificity was determined as (the number of samples that are true negatives/the total number of samples that are true negatives and false positives) × 100. Receiver operating characteristic (ROC) analysis was calculated using GraphPad Prism V8 software (GraphPad Co.). Sensitivity, specificity and ROC analysis were calculated based on RT-PCR results. Each experiment was done twice with each serum sample run in duplicates. Linear regression analysis were performed to infer correlations between antibody levels and sampling time or between the levels of the different antibodies.

## Results

### Expression and production of SARS-CoV-2 proteins

**Figure 1:**
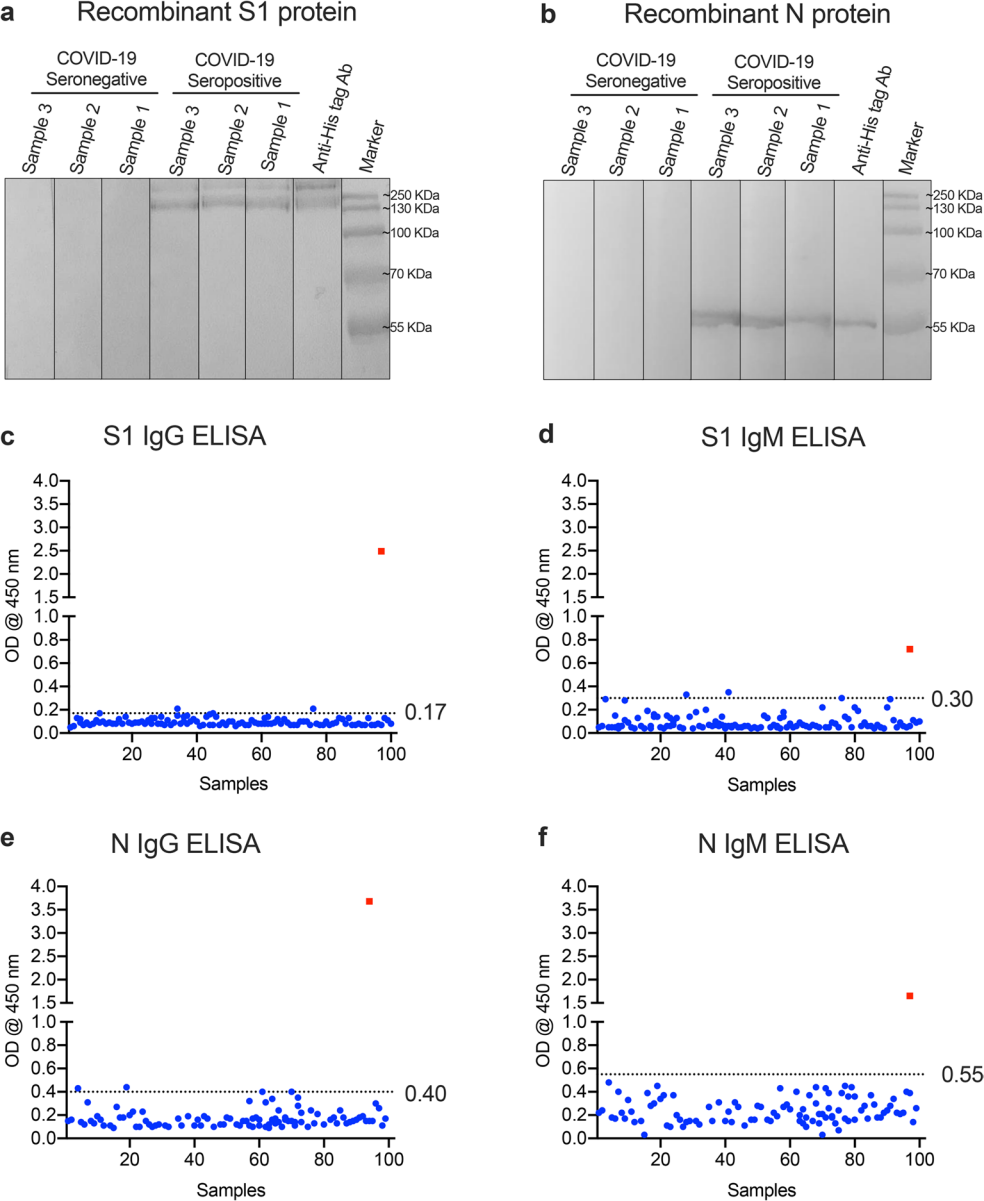
SARS-CoV-2 recombinant proteins and cut-off values for the developed ELISAs. Recombinant SARS-CoV-2 (**a**) S1 or (**b**) N proteins were detected by Western blot using anti-His tag antibodies, known seropositive COVID-19 human samples, or known seronegative COVID-19 human samples. All experiments showed protein bands with expected sizes (~ 110 kDa and ~ 46 kDa for S1 and N, respectively). A 100 serum samples from healthy controls collected before the COVID-19 pandemic were used to determine the cut-off values for (**c**) S1 IgG-ELISA, (**d**) rS1 IgM-ELISA, (**e**) N IgG-ELISA and (**f**) N IgM ELISA. Values were calculated as mean + 3SD. The square is a serologically positive sample from COVID-19 patient. The dotted lines represent the cut-off of each assay.

The S protein of SARS-CoV-2 is a major immunogenic protein and is divided into two subunits; S1 which contains the receptor-binding domain (RBD) and S2 that mediates the fusion with the host membranes^10^. The N protein is another target for most serological assays for CoVs because of its abundant expression^6,7,11^. We and others have shown that both proteins are suitable and comparable for the detection of virus-specific antibodies in MERS-CoV infected patients^7,11^. In this study, we have successfully expressed and purified a His-tagged SARS-CoV-2 N protein and subsequently used it for indirect ELISA development. Recombinant N protein was induced and expressed upon induction with IPTG, and purified on the Ni-NTA affinity chromatography column, while the recombinant S1-His-tagged protein was purchased commercially. Western blot analysis showed that both S1 (~ 110 kDa, Fig. 1a) and N (~ 46 kDa, Fig. 1b) proteins were detected using anti-His antibodies. We also confirmed that only seropositive sera from COVID-19 patients bind specifically to SARS-CoV-2 S1 and N proteins, but not COVID-19 seronegative sera from normal human donors collected before the pandemic (Fig. 1a,b). These data indicate that both S1 and N proteins are antigenically similar to native proteins and able to strongly and specifically detect SARS-CoV-2 antibodies in serum samples.

### Development, optimization, and determination of the cut-off values of the indirect ELISAs

We developed four different types of indirect ELISAs for the testing of anti-SARS-CoV-2 IgM and IgG antibodies using purified SARS-CoV-2 S1 and N proteins as coating antigens. We initially optimized the coating conditions for the ELISA using known SARS‐CoV-2 seronegative and seropositive serum samples and found that the optimal working concentrations of each antigen were 1 μg/ml and 4 μg/ml for recombinant S1 and N proteins, respectively (Supplementary Figure 1). Furthermore, optimal serum dilution was determined using checkerboard titration where the highest OD ratio values of positive to negative samples (P/N) were obtained. After optimization, we tested sera from 100 normal human donors and one serum sample from an RT‐PCR confirmed COVID-19 patient in the developed ELISAs at a dilution of 1:100 to determine the cut-off values (mean + 3 SD). As shown in Fig. 1c–f, the cut-off values were found to be 0.17 (mean = 0.09, SD = 0.03) for S1 IgG-ELISA, 0.30 (mean = 0.09, SD = 0.07) for S1 IgM-ELISA, 0.40 (mean = 0.17, SD = 0.08) for N IgG-ELISA, and 0.55 (mean = 0.24, SD = 0.10) for N IgM-ELISA. Almost all tested samples were below the determined cut-off values suggesting high specificity of the assays.

### Determination of potential cross-reactivity with other CoVs

**Figure 2:**
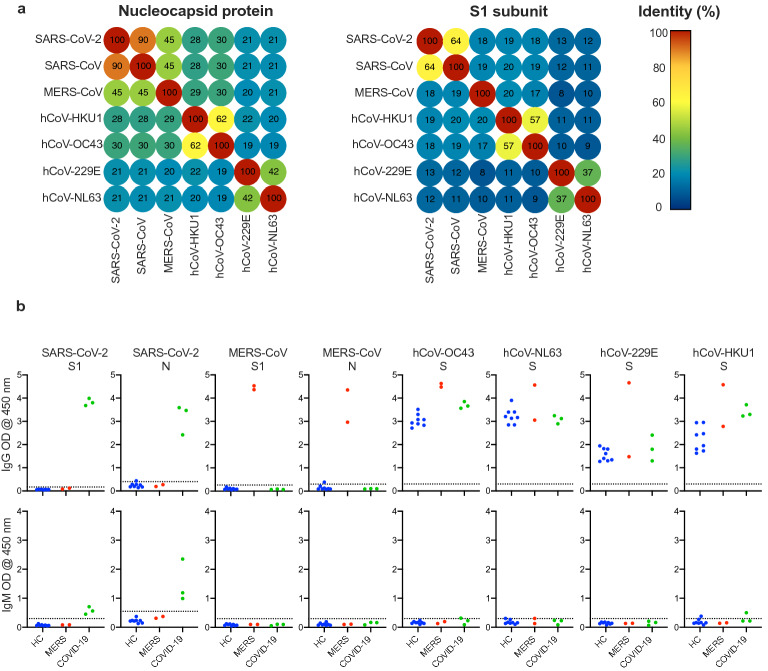
The specificity of the developed ELISAs. (**a**) Sequence homology analysis of SARS-CoV-2 N protein and S1 subunit compared to other human coronaviruses. (**b**) Developed ELISAs were tested for their specificity using sera known to be seronegative for SARS-CoV-2 and MERS-CoV (HC; n = 8), seropositive sera for MERS-CoV (MERS; n = 2) or seropositive sera for SARS-CoV-2 (COVID-19; n = 3). These serum samples were also tested for their reactivity in IgG and IgM ELISAs developed for MERS-CoV S1 and N proteins, as well as full S protein from hCoV-OC43, hCoV-NL63, hCoV-229E, and hCoV-HKU1 viruses. The dotted lines represent the cut-off of each assay. The cut-off values for hCoV-OC43, hCoV-NL63, hCoV-229E, and hCoV-HKU1 ELISAs were set at arbitrary value = blank mean + 3SD.

**Figure 3:**
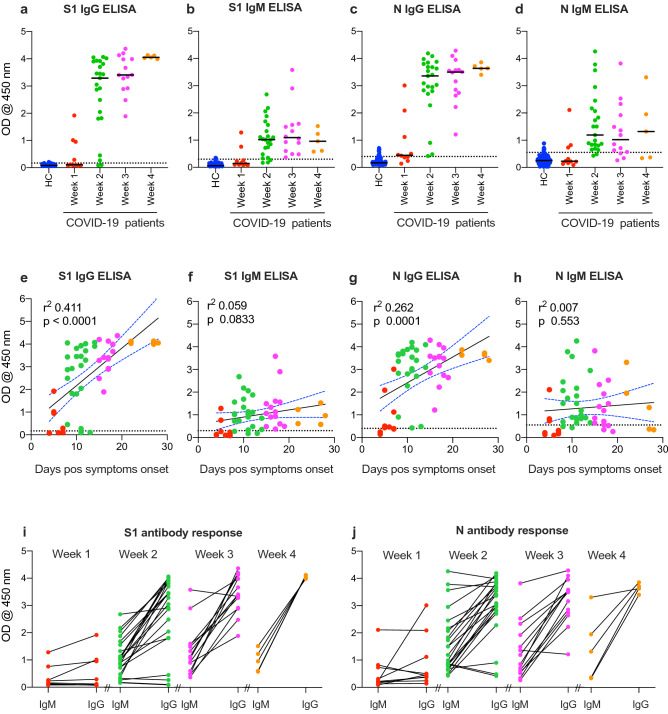
Humoral immune response to COVID-19. Serum samples from healthy controls (n = 125) or COVID-19 patients collected during the 1st week (n = 10), 2nd week (n = 23), 3rd week (n = 14), or 4th week (n = 5) of symptoms-onset were tested for IgG and IgM against SARS-CoV-2 S1 (**a**,**b**) and N (**c**,**d**) proteins using the developed ELISA. The dotted lines represent the cut-off of each assay. Correlation of S1 IgG (**e**), S1 IgM (**f**), N IgG (**g**) and N IgM (**h**) with days after symptom onset. Comparison of IgM and IgG for each patient based on the time of collection for S1 antibodies (**i**) and N antibodies (**j**).

The ability of the developed assay to specifically detect and significantly differentiate SARS-CoV-2 antibodies in patients that might be co-infected with other CoVs was assessed. We first performed sequence homology analysis of SARS-CoV-2 S1 and N compared to other known human CoVs by aligning protein sequences and determining identity. As shown in Fig. 2a, the highest identity of SARS-CoV-2 N protein was with SARS-CoV (90%) as significantly less identity was observed with other human CoVs (19–45%). S1 subunit of SARS-CoV-2 shares only 64% and 57% sequence similarity with SARS-CoV and MERS-CoV, respectively, and 9–37% with other human CoVs. Next, we sought to assess the cross activity of our SARS-CoV-2 S1 and N based ELISA assays. Here, ELISA plates were coated with different capture antigens representing MERS-CoV (S1 and N proteins) and the S protein of the other human CoVs, including hCoV-OC43, hCoV-NL63, hCoV-229E and hCoV-HKU1 at a concentration of 1 μg/ml. Using sera with known seropositivity to MERS-CoV and/or other known human CoVs, we found that our developed SARS-CoV-2 S1 and N-based ELISAs can only detect IgG antibodies from COVID-19 seropositive sera but not those from other tested serum samples that are known to be IgG seropositive for MERS-CoV, hCoV-OC43, hCoV-NL63, hCoV-229E, or hCoV-HKU1 (Fig. 3b). Furthermore, while our SARS-CoV-2 ELISAs only detected IgM from COVID-19 patients, cross-reactivity of these assays with IgM against other CoVs can’t be determined due to the absence of IgM seropositive samples for these viruses. On the other hand, using S1 and N antigens of MERS-CoV only detected antibodies from MERS seropositive samples but not others, confirming the specificity of these ELISAs as we previously reported^7,8^. As expected, using S protein from other human CoVs (hCoV-OC43, hCoV-NL63, hCoV-229E) showed the presence of specific IgG antibodies in almost all tested serum samples suggesting previous exposure to these common cold viruses. Collectively, these data show that our assays can specifically detect and significantly differentiate SARS-CoV-2 specific IgG and IgM antibodies from those against other human CoVs in serum samples.

### Testing of seroconversion

Testing of serum samples collected from another cohort of healthy donors (n = 125) or COVID-19 patients (n = 52) showed that our developed ELISAs could detect both IgG and IgM against both antigens as early as week one post-symptoms-onset (Fig. 3a–d). Our data also show that IgG levels against both antigens increased over time, while IgM levels peaked by week 2 or 3 before starting to decline. Correlation analysis further confirmed these results and showed significant correlation between antibody detection and sampling time post symptoms-onset (Fig. 3e–h). IgG antibodies against S1 (Fig. 3e) or N (Fig. 3g) could be detected in most patients after day 8–10 post symptoms-onset, IgM (Fig. 3f,h) peak levels could only be detected until week 3 before starting to decline (Fig. 3f,h). While some patients produced IgM and IgG against both S1 and N proteins by week 1, many had undetectable levels (Fig. 3i,j). Nonetheless, most patients produced IgM and IgG by week 2 except for few patients who had did not seroconvert or had low levels of IgG (Fig. 3i,j).

### Validation of the developed ELISAs

Based on these data and on the assumption that all RT-PCR positive patients developed humoral response, we sought out to determine the specificity and sensitivity of the developed ELISAs. As shown in Table 1, the specificity of the assays ranged between 91.2–97.6%. The sensitivity, however, was dependent on the sampling time in relevance to disease-onset. During the first-week post symptoms-onset, the sensitivity of IgM and IgG ELISAs ranged between 20–30% and 40–60%, respectively (Table 1). Nonetheless, the sensitivity of the assays increased to 91.3%, 87.0%, 100% and 91.3% for S1 IgG-ELISA, S1 IgM-ELISA, N IgG-ELISA and N IgM-ELISA, respectively by week two. Importantly, while these sensitivity values were maintained at 100% for N IgG-ELISA or increased to 100% for both S1 IgG-ELISA and S1 IgM-ELISA during week three and four post symptoms-onset, N IgM-ELISA’s sensitivity declined. Such results are expected as infected individuals usually develop IgM before IgG, and their IgM titers are anticipated to decline after few weeks compared to IgG titers which elevate and last longer.

**Table 1:** Specificity and sensitivity of the developed ELISAs based on sample time collection.

ELISA	Specificity (%)	Sensitivity (%)			
		Week 1	Week 2	Week 3	Week 4
S1 IgG	97.6	40.0	91.3	100	100
S1 IgM	97.6	20.0	87.0	100	100
N IgG	91.2	60.0	100	100	100
N IgM	94.4	30.0	91.3	78.6	60.0

**Figure 4:**
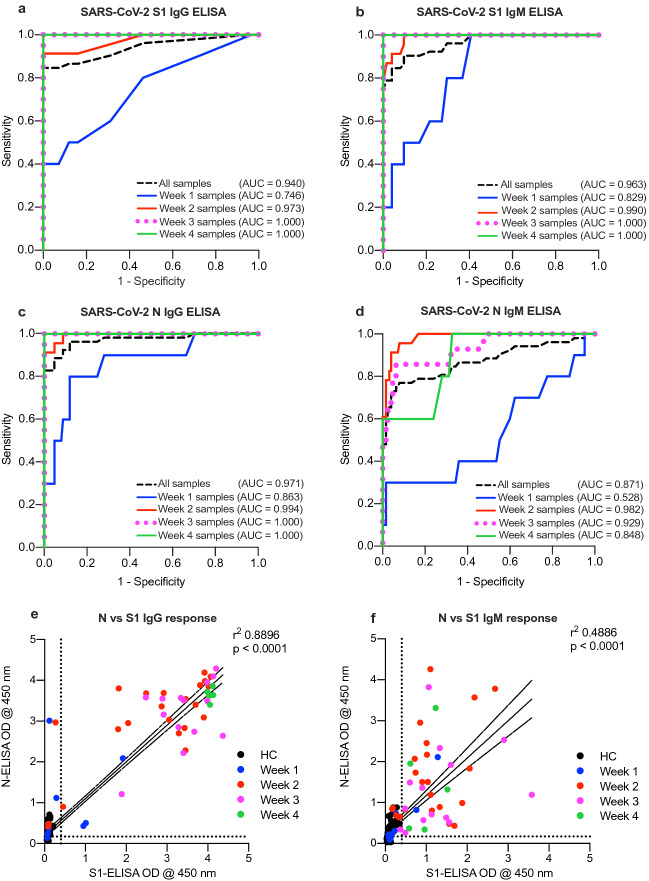
Receiver operating characteristics (ROC) analysis. ROC analysis was applied to positive vs. negative SARS-CoV-2 samples as identified by RT-PCR assay for (**a**) S1 IgG-ELISA, (**b**) S1 IgM-ELISA, (**c**) N IgG-ELISA and (**d**) N IgM ELISA. Serum samples from healthy controls (n = 125) or COVID-19 patients collected during the 1st week (n = 10), 2nd week (n = 23), 3rd week (n = 14), or 4th week (n = 5) of symptoms-onset as well as all COVID-19 samples (n = 52). Correlation of (**e**) S1 and N IgG antibodies and (**f**) S1 and N IgM antibodies.

Next, we conducted a ROC analysis to examine the diagnostic power of each developed assay as shown in Fig. 4a–d. Our analysis showed high accuracy of S1 IgG-ELISA, S1 IgM-ELISA and N IgG-ELISA with overall area under curve (AUC) of 0.938 ± 0.027 (95% CI 0.886–0.990), 0.953 ± 0.021 (95% CI 0.911–0.995) and 0.977 ± 0.015 (95% CI 0.948–1.000), respectively, compared to N IgM-ELISA which showed lower AUC of 0.886 ± 0.037 (95% CI 0.812–0.959) (Supp. Table 1). While the accuracy of these assays in identifying COVID-19 exposed individuals was dependent on the sampling time as it was low when testing samples collected during the first week after symptoms-onset compared to those collected during or after the second week of onset, this is expected as indicated above. Importantly, we observed significantly strong correlation between IgG response against S1 and N (Fig. 4e), suggesting that both assays could be used to evaluate the immune status of infected people or the general population. Similarly, while significant correlation was observed for IgM antibodies against S1 and N (Fig. 4f), IgM antibodies can only be detected during short period of time post infection. Furthermore, high reproducibility was also observed for all assays with very minimal variation (5–10%) in obtained OD values including inter-assay and intra-assay testing conducted on different days or by different individuals (data not shown).

## Discussion

In the current study, we report the development and validation of ELISA-based serological assays for the detection of SARS-CoV-2 specific IgG and IgM antibodies in COVID-19 serum specimens. We showed that our S1 and N-based ELISAs can specifically detect SARS-CoV-2 specific IgG antibodies in sera from COVID-19 patients without cross-reactivity with sera that are seropositive to other human CoVs; including human beta-CoVs such as MERS-CoV, hCoV-OC43, and hCoV-HKU1, as well as alpha-CoVs such as hCoV-NL63 and the hCoV-229. Of note, to confidently rule out the cross-reactivity of our IgM developed assays with other hCoVs, positive sera for these viruses should have been tested but such samples were difficult to obtain because of the high prevalence of these viruses in the population. Therefore, further studies need to consider and address this issue. While we were not able to test cross-reactivity of SARS-CoV seropositive sera due to the unavailability of such samples, cross-reactivity between SARS-CoV and SARS-CoV-2 is expected due to the close phylogenetic relationship and the higher genome and protein sequences identity between SARS-CoV and SARS-CoV-2 compared to other human CoVs (Fig. 2a). However, it is of note that S1 subunit shows more virus specificity and divergence among the different CoVs compared to full-length S protein and subsequently less cross-reactivity^12–14^. On the other hand, cross-reactivity was clearly observed between COVID-19 and SARS-CoV seropositive serum samples against either SARS-CoV-2 or SARS-CoV N proteins^13,14^.

Furthermore, using the developed ELISAs, we evaluated the production of SARS-CoV-2 specific IgG and IgM antibodies in a cohort of hospitalized COVID-19 patients (n = 52), including samples collected during the 1st week (n = 10), 2nd week (n = 23), 3rd week (n = 14) or 4th week (n = 5) of symptoms-onset. Our analysis showed that SARS-CoV-2 IgM or IgG specific antibodies for either SASR-CoV-2 S1 or N antigens can be detected virtually in all RT-PCR confirmed COVID-19 patients in this study. We showed that both virus-specific IgG and IgM can be detected as early as one week after disease-onset but significantly increased by week two and three, with IgG persisting through week four (last time point in our study) compared to IgM which peaked by week 2 or 3 before declining. This increase in IgG over time and the decline in IgM antibodies by week 4 are consistent with some recent reports^15–18^. Most patients seroconverted to IgG against both antigens (S1 and N) by week 2, and both antibodies significantly correlated with days post symptoms-onset.

To be able to use the developed assays for large scale serosurveys, we determined the cut-off values, specificity, and sensitivity of the different developed ELISAs. While our analysis showed that the cut-off values were 0.17 for S1 IgG-ELISA and 0.30 for S1 IgM-ELISA, the cut-off values for the N based ELISAs were found to be 0.40 and 0.55 for IgG and IgM antibodies, respectively. Almost all seronegative samples were below the determined cut-off values, indicating the high specificity of the assays. Our ROC analysis also demonstrated the powerful diagnostic performance of the developed assays.

The fact that all RT-PCR confirmed COVID-19 patients included in this study developed virus-specific antibody responses should be reassuring especially that antibodies were detected as early as week one. Although it has not been proven whether the mounted anti-SARS-CoV-2 antibody response could offer long-lasting protection against COVID-19, such responses are likely to be associated with protection from reinfection. Reinfection in humans has not been reported in SARS-CoV or MERS-CoV, and antibody responses against these two viruses were reported to last for up to 3 years^19,20^. Interestingly, a recent report examined the possibility of SARS-CoV-2 reinfection in non-human primates and showed that reinfection was unlikely after the induction of antibody responses^21,22^. Nevertheless, the possibility of reinfection in humans is a pressing question that warrants further investigations. Additionally, it has been shown that convalescent plasma containing high titer of SARS-CoV-2–specific IgG antibodies improved the clinical outcomes of severe COVID-19 cases^23^. The assays we presented here would be of great utility not only to conduct such studies but also to examine the longevity of the mounted antibody responses against SARS-CoV-2 infection, which is critical for vaccine development efforts. Such serological assays should be able to address these questions in the near future. The early detection of specific antibodies in COVID-19 patients also highlights the diagnostic importance of these assays especially in asymptomatic as well as mild cases that usually present late to hospitals or go undetected.

Some seropositive COVID-19 sera were also found positive to other low pathogenic human CoVs, which may indicate that previous infections with other CoVs provide no immunity, at least in our cohort of COVID-19 patients. Interestingly, a recent study attempted to understand why SARS-CoV-2 infected children developed less severe symptoms compared to adults, suggested a possible cross-protection due to previous infections with circulating common cold CoVs, mostly through virus-specific T cell responses^24^. While we cannot confirm this suggestion here since the age range of the COVID-19 patients in our study was between 24 to 75 years and we only examined humoral immune responses, future studies clearly need to investigate this possibility further.

Few serological assays have been reported thus far and most of them use the full S protein, S1 subunit or the RBD as capture antigens^9,15–17,25^. While these assays show high sensitivity and specificity rates, the use of the S1 or the RBD alone may result in missing cases or give a less accurate estimation of the mounted antibody response since high levels of antibodies are generated to areas outside S1 or RBD^26^. Additionally, as it mediates binding and entry into cells and being a target for neutralizing antibodies, the S protein is under continuous selective pressure, which makes it more prone to acquire mutations that might affect the accuracy of S-based serological assays^27^. In our assays, to overcome the aforementioned issues we included N-based ELISA in addition to S1 and found them complementary to each other with both showing high sensitivity and specificity. Another reason to include N-based ELISA in the serological testing algorithm is its relatively small size and lack of glycosylation sites, which makes it easy to clone and produce in prokaryotic expression systems, especially in resource-limited settings^4^. Importantly, our data show that IgG antibodies against both S1 and N proteins show significant and strong correlation. Furthermore, it is now evident that asymptomatic infections occur and could play an important role in virus spread^28–30^. Thus, the ability to detect asymptomatic or mild cases is crucial for epidemiological investigations^9,16^. Therefore, we believe that using both S1 and N in serological testing would capture as many potential SARS-CoV-2 positive cases as possible than using any of them alone. This is of great importance amid the current rapid and continuing spread of SARS-CoV-2 and the need for a quick and efficient method for contacts and cases tracing.

The current standard method for the detection of SARS-CoV-2 relies on the detection of the viral RNA by RT-PCR. Although this highly sensitive method can effectively detect SARS-CoV-2 infection during the acute infection phase, RT-PCR is time-consuming and has a limited detection rate of the virus beyond week 3 after symptoms-onset^31,32^. Some of these issues could be addressed by the availability of validated serological assays. Moreover, the development of serological assays is an essential step for the understanding of the epidemiology of SARS-CoV-2 infection. Of note, while our study reports validated ELISA assays, we have not assessed virus neutralization activities of detected antibodies. However, recent studies have shown a positive correlation between high titers of IgG antibodies detected by ELISAs with neutralizing antibodies^25^.

We believe that our assays are well-validated, highly specific, sensitive, and can be used for serosurveys to inform us about the extent of the current spread of COVID-19 pandemic in the population. Such studies are also important for a better understanding of the nature of the immune response to SARS-CoV-2, and the true estimate of the attack and infection fatality rates in different human populations.

## Supplementary information

Supplementary Information.
