# Supplementary material for: SARS-CoV-2 S1 and N-based serological assays reveal rapid seroconversion and induction of specific antibody response in COVID-19 patients
Source: Sci Rep. 2020 Oct 6;10:16561. doi: 10.1038/s41598-020-73491-5 (PMC7538990; doi:10.1038/s41598-020-73491-5)
Supplement: Supplementary file 1 — Supplementary Information. [file 41598_2020_73491_MOESM1_ESM.pdf]

**SARS-CoV-2 S1 and N-based serological assays reveal rapid seroconversion and induction of specific antibody response in COVID-19 patients**

Abdullah Algaissi[1,2,3,#], Mohamed A Alfaleh[1,4,#], Sharif Hala[5,6,#], Turki S Abujamel[1,7], Sawsan S Alamri[1,8], Sarah A Almahboub[1], Khalid A Alluhaybi[1,4], Haya I Hobani[1], Reem M Alsulaiman[1], Rahaf H AlHarbi[1,9], M-Zaki ElAssouli[1], Rowa Y Alhabbab[1,7], Ahdab A AlSaieedi[1,7], Wesam H Abdulaal[8], Afrah A AL-Somali[10], Fadwa S Alofi[11], Asim A Khogeer[12], Almohanad A Alkayyal[13], Ahmad Bakur Mahmoud[14], Naif AM Almontashiri[15], Arnab Pain[5,16,17], Anwar M Hashem[1,18,*]

[1] Vaccines and Immunotherapy Unit, King Fahd Medical Research Center; King Abdulaziz University, Jeddah, Saudi Arabia

[2] Department of Medical Laboratories Technology, College of Applied Medical Sciences, Jazan University, Jazan, Saudi Arabia

[3] Medical Research Center, Jazan University, Jazan, Saudi Arabia

[4] Faculty of Pharmacy, King Abdulaziz University, Jeddah, Saudi Arabia

[5] Pathogen Genomics Laboratory, Division of Biological and Environmental Sciences and Engineering (BESE), Thuwal, Saudi Arabia

[6] King Abdullah International Medical Research Centre, King Saud University for Health Sciences, Ministry of National Guard Health Affairs, Jeddah, Saudi Arabia

[7] Department of Medical Laboratory Technology, Faculty of Applied Medical Sciences, King Abdulaziz University, Jeddah, Saudi Arabia

[8] Department of Biochemistry, Faculty of Science, King Abdulaziz University, Jeddah, Saudi Arabia

[9] Department of Biology, faculty of science, King Abdulaziz university, Jeddah , Saudi Arabia

[10] Infectious Diseases Department, King Abdullah medical complex, Jeddah, Saudi Arabia

[11] Infectious Diseases Department, King Fahad Hospital, Almadinah Almunwarah, Saudi Arabia

[12] Plan and Research Department, General Directorate of Health Affairs Makkah Region, Ministry of Health, Makkah, Saudi Arabia

[13] Department of Medical Laboratory Technology, University of Tabuk, Tabuk, Saudi Arabia

[14] College of Applied Medical Sciences, Taibah University, Almadinah Almunwarah, Saudi Arabia

[15] Center for Genetics and Inherited Diseases, Taibah University, Almadinah Almunwarah, Saudi Arabia

[16] Research Center for Zoonosis Control, Hokkaido University, N20 W10 Kita-ku, Sapporo, Japan

[17] Nuffield Division of Clinical Laboratory Sciences (NDCLS), University of Oxford, Oxford, OX3 9DU, UK

[18] Department of Medical Microbiology and Parasitology, Faculty of Medicine, King Abdulaziz University, Jeddah, Saudi Arabia

[#] equal contribution

* **Corresponding author:** Anwar M Hashem; Faculty of Medicine and King Fahd Medical Research Center, King Abdulaziz University, P.O.Box 80205, Jeddah 21859, Saudi Arabia. Tel. +966 (12) 6400000 ext. 21033, E-mail: amhashem@kau.edu.sa.

**Key words:** SARS-CoV-2; COVID-19; ELISA, Antibodies; Serology

**Running title:** ELISAs for COVID-19 antibodies testing

**Supplementary Table 1. Area under the ROC curve (AUC) for the different developed ELISAs based on sample time collection.**

| ELISA | Samples | AUC ± SD | 95% CI | P value |
|---|---|---|---|---|
| S1 IgG | All samples | 0.940 ± 0.024 | 0.892 - 0.986 | <0.0001 |
| | Week 1 samples | 0.746 ± 0.091 | 0.567 - 0.925 | 0.0099 |
| | Week 2 samples | 0.973 ± 0.020 | 0.935 - 1.000 | <0.0001 |
| | Week 3 samples | 1.000 ± 0.000 | 1.000 - 1.000 | <0.0001 |
| | Week 4 samples | 1.000 ± 0.000 | 1.000 - 1.000 | 0.0002 |
| S1 IgM | All samples | 0.963 ± 0.014 | 0.935 - 0.990 | <0.0001 |
| | Week 1 samples | 0.829 ± 0.052 | 0.727 - 0.931 | 0.0006 |
| | Week 2 samples | 0.990 ± 0.007 | 0.977 - 1.000 | <0.0001 |
| | Week 3 samples | 1.000 ± 0.000 | 1.000 - 1.000 | <0.0001 |
| | Week 4 samples | 1.000 ± 0.000 | 1.00 0 -1.000 | 0.0002 |
| N IgG | All samples | 0.971 ± 0.015 | 0.942 - 1.000 | <0.0001 |
| | Week 1 samples | 0.863 ± 0.065 | 0.736 - 0.990 | 0.0001 |
| | Week 2 samples | 0.994 ± 0.005 | 0.985 - 1.000 | <0.0001 |
| | Week 3 samples | 1.000 ± 0.000 | 1.000 - 1.000 | <0.0001 |
| | Week 4 samples | 1.000 ± 0.000 | 1.000 - 1.000 | 0.0002 |
| N IgM | All samples | 0.871 ± 0.035 | 0.803 - 0.940 | <0.0001 |
| | Week 1 samples | 0.528 ± 0.111 | 0.311 - 0.746 | 0.7655 |
| | Week 2 samples | 0.982 ± 0.009 | 0.965 - 1.000 | <0.0001 |
| | Week 3 samples | 0.929 ± 0.038 | 0.854 - 1.000 | <0.0001 |
| | Week 4 samples | 0.884 ± 0.067 | 0.753 - 1.000 | 0.0037 |

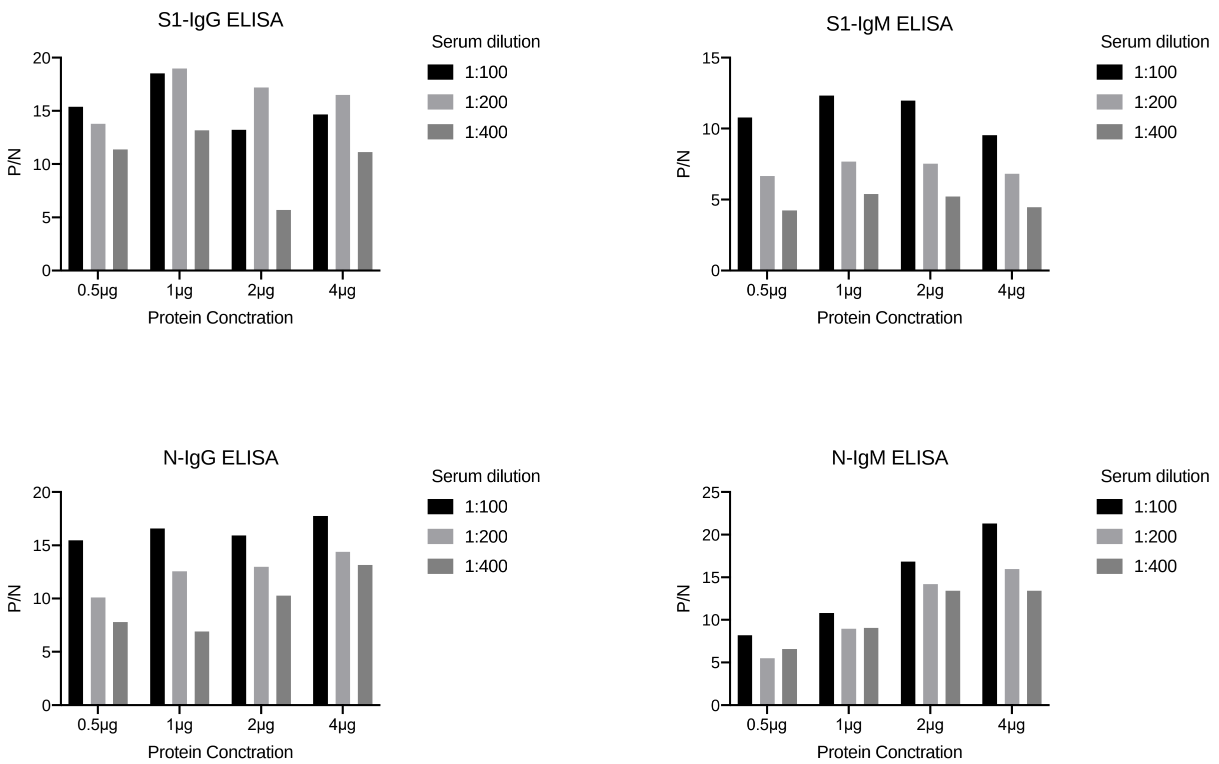


**Supplementary Figure 1.** checkerboard titration to determine the optimium antigen concentration and dilution of serum samples in the developed SARS-CoV-2 ELISAs
